# Cyanobacterial elicitor enhances the biomass of *Mentha piperita* L. and improves the production of high-value rosmarinic acid under in vitro culture of apical meristem

**DOI:** 10.1186/s12870-024-04876-1

**Published:** 2024-03-15

**Authors:** Zeinab Shariatmadari, Somayeh Zarezadeh, Hossein Riahi, Ali Akbar Ghotbi-Ravandi, Mehri Seyed Hashtroudi, Ensiyeh Shahroudi

**Affiliations:** 1 Department of Plant Sciences and Biotechnology, Faculty of Life Sciences and Biotechnology, Shahid Beheshti University, Tehran, Iran; 2 Ocean Science Department, Iranian National Institute for Oceanography and Atmospheric Sciences, Tehran, Iran; 3 Department of Plant Biology, Tarbiat Modares University, Tehran, Iran

**Keywords:** Bioelicitor, Cyanobacteria, In vitro propagation, Peppermint, Rosmarinic acid

## Abstract

**Background:**

Rosmarinic acid (RA), like other phenolic compounds, is sources of antioxidants and anti-inflammatory agents in medicinal plants. In vitro culture of plants can improve the medicinal plants’ metabolite profile and phenolic compound quantity. To date, various methods have been proposed to increase this medicinal metabolite in plants, among which the use of bioelicitors can be mentioned. In the present study, a native isolate of heterocystous cyanobacteria, *Nostoc spongiaeforme* var. *tenue* ISB65, was used to stimulate the production of biomass and content of RA in *Mentha piperita* L. (peppermint) grown in vitro from apical meristem. *Mentha piperita* L. explants were inoculated in half strength Murashige and Skoog (1/2 MS) medium containing cyanobacterial lysate (CL). After 50 days of culturing, the growth indices, the content of photosynthetic pigments, and RA in control and treated plants were measured.

**Results:**

CL inoculation resulted in a significant enhancement in the vegetative growth indices of peppermint, including root and shoot length, plant biomass and leaf number. The content of photosynthetic pigments also increased in cyanobacteria-treated plants. Inoculation with CL increased the RA content by 2.3-fold, meaning that the plants treated with CL had the highest RA content (7.68 mg. g^− 1^ dry weight) compared to the control (3.42 mg. g^− 1^ dry weight). Additionally, HPLC analysis revealed the presence of several auxins in CL.

**Conclusions:**

The presence of auxins and the chemical content of CL such as K^+^ and Ca^2+^, as regulators of metabolic pathways and molecular activities of cells, may be responsible for the enhanced growth and phenolic compounds of plants under tissue culture conditions. An improvement in RA content in the tissue culture of medicinal plants treated with CL was reported for the first time in this investigation.

## Background

Peppermint (*Mentha piperita* L.) is a perennial plant belonging to the family Labiatae. This hybrid, a cross between *Mentha spicata* L. and *Mentha aquatica* L., is considered native to temperate regions of the world, especially Europe, North America, and North Africa [1]. *M. piperita* has antibacterial, antiviral, antioxidant, antispasmodic, anti-bloating, and anti-asthma properties [2, 3]. Due to these properties, peppermint is commonly used in the food, perfumery, health, and cosmetic manufacturing industries [4]. This plant contains various medicinal metabolites, such as menthol, menthone, menthofuran, and phenolic compounds including rosmarinic acid (RA) [1, 5]. The synthesis of RA is known to be one of the major defense mechanisms of plants against free radical damage [6, 7]. Its tannin-like properties also protect the plant against pathogens and herbivores [8].

The presence of RA has been reported in several higher plant families such as Lamiaceae, Apiaceae, Boraginaceae, as well as some ferns and hornworts [9, 10]. This compound, forms when caffeic acid and 3,4-dihydroxyphenylacetic acid combine in an ester bond, exhibits antioxidant, antimicrobial, antiviral, and anti-inflammatory activities [11, 12]. Moreover, it has anti-mutagenic ability, as well as cardioprotective, hepatoprotective, and photoprotective activity [8].

Given that medicinal metabolites are present in small amounts in plants and their extraction is time-consuming and expensive process, strategies that enhance these metabolites in medicinal plants are of great importance. In recent years, approaches such as plant tissue culture and gene editing have been developed to increase the amount of medicinal metabolites [13, 14]. In addition to increasing the amount, mentioned methods can also improve the quality of these valuable metabolites. The expansion of these methods is important, especially in areas where, due to the climate conditions, the cultivation and production of medicinal plants is difficult.

In recent years, plant cell and tissue culture methods have been suggested to boost the accumulation of phenolic compounds in medicinal plants [13, 15–17]. Furthermore, the in vitro culture of medicinal plants is useful for obtaining unified plant biomass on a large scale with a guaranteed aroma and metabolite quality [3]. According to Abdel Rahman et al., plant tissue culture is an effective technique for producing RA in *Ocimum basilicum* L. and *Melissa officinalis* L., and they observed an enhancement in its content in undifferentiated cell cultures [18]. Other studies have confirmed similar results in sweet basil (*Ocimum basilicum* L.) tissue culture [19]. Moreover, previous investigations have reported the optimization of RA production by callus culture of medicinal plants, such as *Salvia nemorosa* L. and *Satureja hortensis* L. [20, 21]. 

Based on the recent studies, various elicitors have shown improved production of phenolic compounds in various economic and medicinal plants [15]. Elicitors are the key factors that induce plant defensive responses followed by an increase in targeted secondary metabolites [22]. For example, the use of bioelicitors such as yeast, mycorrhizae, cyanobacteria, and algae to increase phenolic compounds in economic plants has been reported [23–27]. The increase in RA content in medicinal plants as a result of abiotic elicitor application has also been reported [22, 25].

Cyanobacteria are the simplest group of photosynthetic organisms with a great capacity to stimulate plant growth [28–30] by producing plant growth regulators, improving plant mineral nutrition, and fixation of atmospheric nitrogen (N_2_) which makes them efficient bioelicitors [29, 31, 32]. In addition to inducing plant vegetative growth, cyanobacteria can optimize targeted secondary metabolite production in medicinal plants. Several factors can affect secondary metabolite accumulation in the tissue culture of plants, including phytohormones and microelement levels [8]. Therefore, cyanobacteria can also optimize the production of valuable plant metabolites in different ways.

Despite the existence of numerous reports about the regulatory effect of elicitors on phenolic compounds of plants, cyanobacteria have received less attention as bioelicitors. Due to limited reports about the impact of cyanobacteria on the tissue culture of medicinal plants as bioelicitors, the current study aimed to evaluate the growth-promoting potential of the heterocystous cyanobacterium *Nostoc spongiaeforme* var. *tenue* ISB65 on *Mentha piperita* L. under tissue culture conditions. The effects of the inoculation were assessed based on various growth indices, photosynthetic pigment contents, and RA contents of plant biomass. In addition, the key factors affecting plant growth and metabolite quantity in cyanobacteria-treated plants were investigated.

## Methods

### Preparing cyanobacterial lysate (CL)

Following soil collection from *Plantago major*’s bed in Mazandaran Province, Iran, by Chookalaii et al. [26], isolated and axenic cultures of the heterocystous cyanobacterium *N. spongiaeforme* var. *tenue* ISB65 were prepared after continuous subcultivation in agar plates of BG-110 medium and incubation under 12 h artificial light/12 h dark cycles at 25 °C [33].

Identification of cyanobacterium was performed using a BH-2 light microscope (Olympus). Cyanobacteria paste prepared after harvesting the culture at the end of the exponential growth phase (four weeks after incubation). CL was prepared according to Nascimento et al.’s modified method [34]. The sample was frozen for 24 h at -20 °C, then defrosted at room temperature (three times), followed by ultrasonication (FAPAN 300UPL, Iran) for 10 min (20 s cycles with 40 s resting time). Then, the obtained lysate was centrifuged at 12,000 rpm for 10 min, and the supernatant was used as the CL. Finally, CL was sterilized through filtration by using a 0.2 μm filter before being added to the tissue culture media.

### Shoot micropropagation

After obtaining *M. piperita* L. explants from ACECR (Iranian Institute of Medicinal Plants), samples were disinfected by soaking in 1% sodium hypochlorite for 10 min, rinsing with sterilized distilled water, immersion in 70% ethanol for 1 min, and rinsing with sterilized distilled water for five times. Disinfected apical meristems were cultivated on solid Murashige and Skoog (MS) medium containing 7 g.L^− 1^ agar, 15 g.L^− 1^ sucrose, and 2 gr. L^− 1^ activated charcoal. After four weeks, the apical meristems of in vitro cultivated plants were utilized for the experiment.

Thirty jars each containing 40 mL MS medium was prepared for each control and CL treatment. In addition, 2 gr. L^− 1^ CL was added to the pre-autoclaved CL treatment medium. Each jar was cultured with 8 apical meristems of *M. piperita* and randomly settled in a culture chamber under 12 h artificial light/12 h dark cycles at 25 °C.

### Measurement of growth parameters

The in vitro cultivated plants were harvested after 50 days of incubation for morphological, physiological, and metabolic evaluations. Quantitative indices related to the plants growth, including dry and fresh biomass, shoot and root length, leaf number and ramification were determined. Fresh weights were measured by weighing the root and shoot for each plant, separately. Root and shoot of each plant was oven dried at 38 °C for three days to measure dry weights. Further analysis such as pigment content and extraction were performed using fresh leaves.

### RA extraction and analysis

RA was extracted based on the procedure provided by Phatak and Heble [35]. In addition, HPLC analysis was conducted on a Waters liquid chromatography apparatus consisting of a Waters separations module 2695 (USA) and a Waters dual absorbance detector 996 (USA). To load the samples, automatic injection was carried out using a 100 µL volume loop. Data were acquired and integrated by using Millennium 32 software. A 25 cm×4.6 mm Eurospher 100-5 C_18_ column (25 cm×4.6 mm×5 μm) provided by KNAUER (Berlin, Germany) was utilized for the chromatographic assay. Furthermore, elution was performed in isocratic mode with acetonitrile as solvent A and water as solvent B at a flow rate of 1 mL.min^− 1^. The peaks were monitored by using a UV detector at 255 nm. It is worth noting that the injection volume was considered 20 µL, and the temperature was maintained at 25 °C.

### Photosynthetic pigment content

For this purpose, 5 mg of plant leaves was powdered in liquid nitrogen and suspended in 20 mL of 80% acetone. Then, the suspension was centrifuged for 10 min, the supernatant of which was withdrawn following centrifugation at 6000 rpm. After filtration, the absorbance of samples was measured at 470, 646, and 663 nm using Unico 2100 Vis Spectrophotometer. Finally, the carotenoid and chlorophyll contents were calculated according to Arnon [36].

### Extraction and HPLC analysis of auxins

The auxin standards indole 3-acetic acid (IAA) and indole-3-butyric acid (IBA) were purchased from Duchefa Biochemie (Haarlem, Netherlands), and indole-3-propionic acid (IPA) was obtained from Merck Chemicals (Darmstadt, Germany). All solvents were of HPLC grade and were provided by Samchung Chemical Co. (Seoul, Korea). Ultrapure water used in all experiments was obtained from a Millipore Direct Q UV-3 system. The experimental procedures for extraction, identification, and quantification of endogenous auxins in the studied cyanobacterium were performed as described by Seyed Hashtroudi et al. [37]. The algal biomass, initially freeze-dried in an Operon bench top freeze dryer (FDB-5503), was extracted by methanol-water 80:20 in an ultrasonic bath (Bandelin SONOREX DIGITEC DT 103 H, Germany) for 30 min at 20 °C. The suspension was centrifuged at 6.728×g for 10 min, and then filtered through a 0.45-µm PTFE syringe filter, and the final volume was reduced to 500–1000 µL under a stream of N_2_. The stock standard solution of target auxins, IBA, IAA, and IPA was provided by dissolving 1 mg of auxins in 10 mL of methanol-water (80:20, v/v). The subsequent concentrations required to plot the calibration curve were made by serial dilution of the initial standard. All standards and extracts were stored at 0–4 °C. Chromatographic analysis of auxins was conducted on an Agilent 1200 series HPLC system. Instrument control and processing were obtained by HPLC with ChemStation software.

A Eurosphere RP-column was applied for separation and measurement of auxins. The injection volume of samples was 20 µL. Three replications and three runs were applied for experiment. To calculate the recovery of the analytes, a certain amount of the standard was added to the cyanobacterial sample, and the extraction and quantification steps were performed exactly the same as those for the real samples. Finally, the auxins were quantified by using the calibration curves created by plotting the peak areas versus six concentrations of three intended auxins. Table 1 shows the amount of endogenous auxins (IAA, IBA, and IPA) in the extract of *N. spongiaeforme* var. *tenue* ISB65.

**Table 1:** Auxin content of cyanobacterial extracts (ng.g^− 1^DW)

Taxon	Indole 3-acetic acid	Indole 3- butyric acid	Indole 3- propionic acid
*Nostoc spongiaeforme* ISB65	35.3	4.2	24.7

### Evaluating the chemical content of cyanobacterium

To obtain the chemical composition of the cyanobacterium, the amounts of total nitrogen (N), nitrite (NO_2_^−^), nitrate (NO_3_^−^), ammonium (NH_4_^+^), sulfate (SO_4_^2−^), phosphate (PO_4_^3−^), carbonate (CO_3_^2−^), magnesium (Mg^2+^), calcium (Ca^2+^), sodium (Na^+^), and potassium (K^+^) ions were determined at the Institute of Arian Fan Azma, Iran, following the methods summarized in Table 2. Each test was carried out in three replications, and the averages of the obtained amounts were provided.

**Table 2:** Chemical content of cyanobacteria

	Analytical Method	*Nostoc spongiaeforme* ISB65
Total N (mg.L^− 1^)	Macro kjeldahl	35.00
NO_2_^−^ (mg.L^− 1^)	Colorimetric	0.60
NO_3_^−^ (mg.L^− 1^)	Ultraviolet Spectrophotometric	9.40
NH_4_^+^ (mg.L^− 1^)	Nesslerization	5.00
Phosphate (mg.L^− 1^)	Vanadomolybdophosphoric acid colorimetric	9.00
CO_3_^2−^ (mg.L^− 1^)	Titrimetric	0.00
HCO_3_^−^ (mg.L^− 1^)	Titrimetric	30.00
Na^+^ (mg.L^− 1^)	Flame Emission Photometric	1.50
Mg^2+^ (mg.L^− 1^)	EDTA Titrimetric	5.00
Ca^2+^ (mg.L^− 1^)	EDTA Titrimetric	7.00
K^+^ (mg.L^− 1^)	Flame Emission Photometric	0.11
EC (µS cm^− 1^)	Platinum Electrode	15.00
pH	Electrometric	6.02

### Data analysis

To analyze the data, which were obtained from three biological replicates and are expressed as the mean ± SE, one-way ANOVA in SPSS 16.0 was utilized. The mean values were separated by applying the Tukey HSD test at *P* < 0.05.

## Results

### Measured parameters in plants

**Fig. 1:**
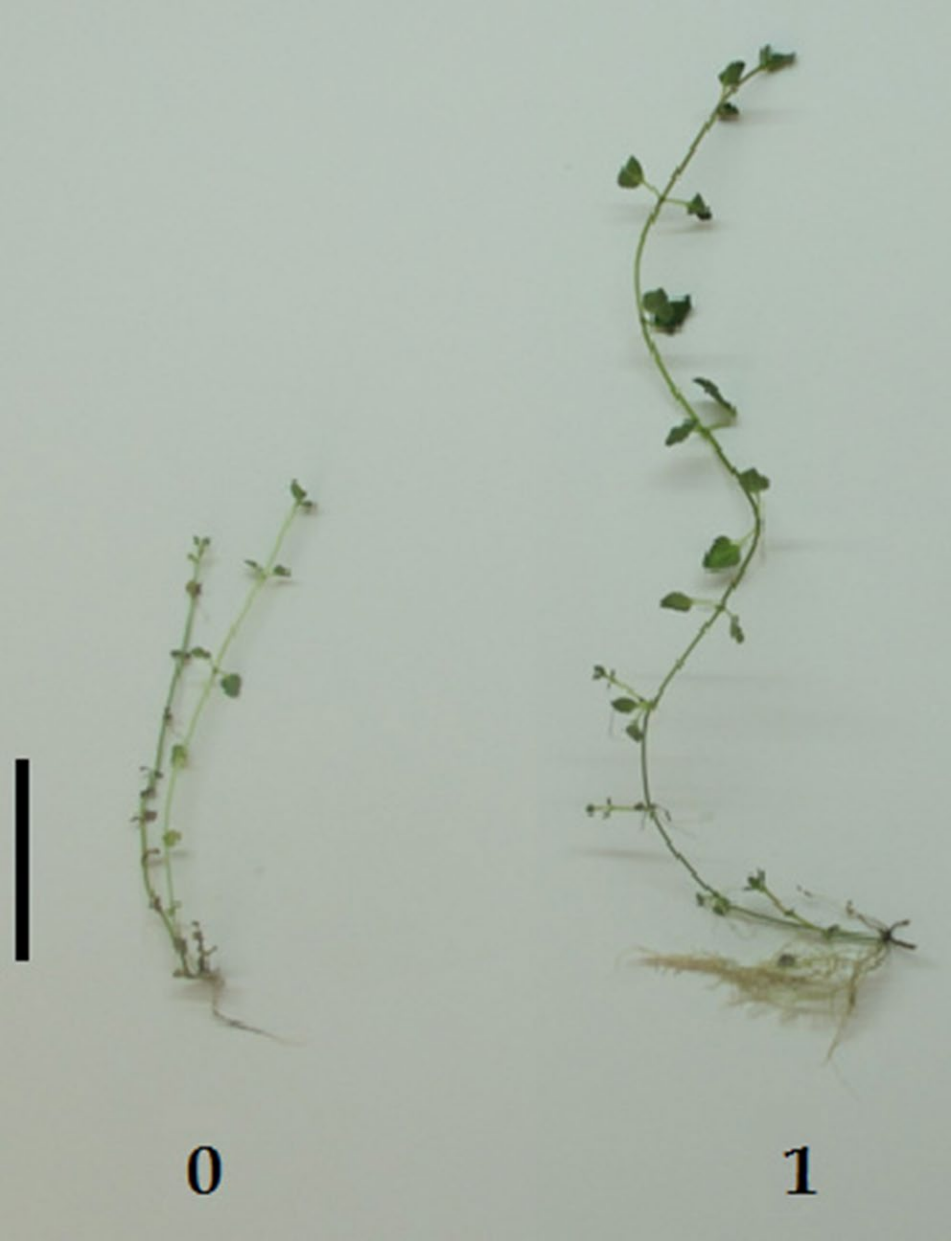
Comparison of plants in growth factors, 0. Control (Cultured in MS medium), 1. Plants treated with *Nostoc spongiaeforme* ISB65 (Cultured in MS + CL), (bar = 5 cm)

Inoculation of peppermint by CL led to a remarkable increase in plant growth indices (Fig. 1), which were significant in parameters of shoot and root length (144% and 48%, respectively, *P* < 0.05), leaf number (100%, *P* < 0.05), fresh weight of roots (87%, *P* < 0.05), and dry biomass of shoots (93%, *P* < 0.05) (Fig. 1; Table 3).

**Table 3:** Effect of CL on growth indices of *Mentha piperita* L. in tissue culture after 50 days of planting (Mean ± SE)

Characters	Control	*Nostoc spongiaeforme* ISB65
Shoot length (cm)	11.00 ± 0.01	26.90 ± 1.02 *
Root length (cm)	3.60 ± 0.20	5.35 ± 0.00 *
Fresh weight of shoot (g)	0.39 ± 0.29	0.55 ± 0.00
Dry weight of shoot (g)	0.16 ± 0.00	0.31 ± 0.01*
Fresh weight of root (g)	0.08 ± 0.01	0.15 ± 0.00 *
Dry weight of root (g)	0.04 ± 0.00	0.06 ± 0.00
Leaf number (no.)	17.33 ± 0.66	34.66 ± 0.66 *
Ramification (no.)	2.00 ± 0.00	2.33 ± 0.33

^* significant at the 0.05 level^

In addition to the growth parameters, the application of CL in tissue culture led to a slight raise in chlorophyll *a*, *b*, and total carotenoids in *M. piperita* in comparison to the controls. However, no significant difference was obtained regarding photosynthetic pigment content between the treated and control plants (Table 4).

**Table 4:** Effect of CL on photosynthetic pigments of *Mentha piperita* L. in tissue culture after 50 days planting (Mean ± SE)

Characters	Control	*Nostoc spongiaeforme* ISB65
Chl a	5.24 ± 0.02	5.67 ± 0.26
Chl b	1.17 ± 0.00	1.34 ± 0.04
Total Carotenoid	3.03 ± 0.01	3.23 ± 0.12

^* significant at the 0.05 level^

**Fig. 2:**
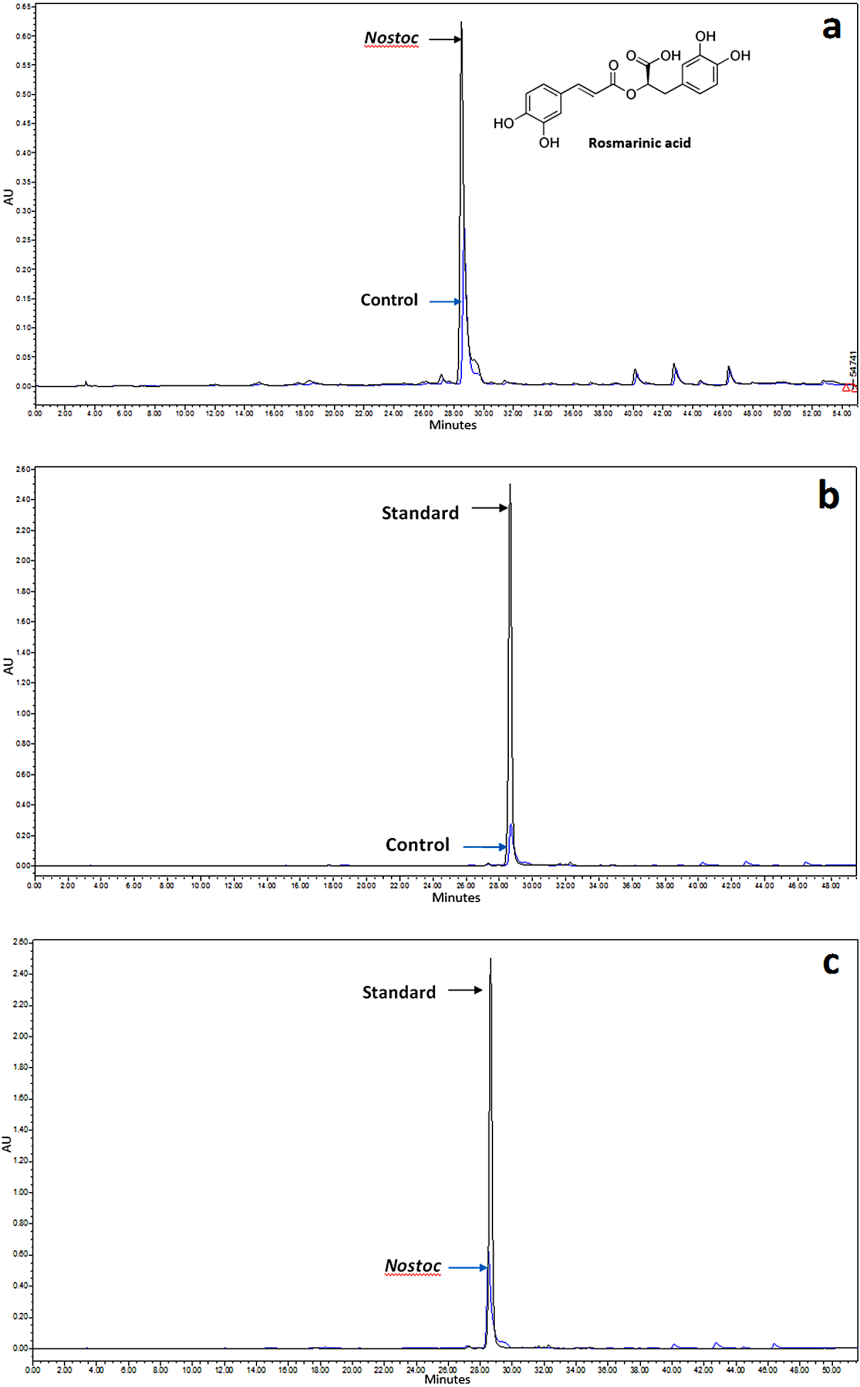
HPLC chromatograms of the extracts from **(a)** control and *Nostoc*-treated plants in tissue culture of *Mentha piperita* L., **(b)** rosmarinic acid standard solution and control plant extract, and **(c)** rosmarinic acid standard solution and *Nostoc*-treated plant extract

The RA concentrations in the treated and control plant biomasses are provided in Table 5. Based on the results, the average content of RA in the plants treated with CL were 7.68 mg. g^− 1^ dry weight, while in control it was 3.42 mg. g^− 1^ dry weight. In other words the amount of RA enhanced by 125% (almost 2.3-fold) (Fig. 2; Table 5).

**Table 5:** Rosmarinic acid content of plant extracts (Mean ± SE)

Treatment	Rosmarinic acid content (mg.g^−1^DW)
Control	3.42 ± 0.08
*Nostoc spongiaeforme* ISB65	7.68 ± 0.21

### Measured parameters in the CL

**Fig. 3:**
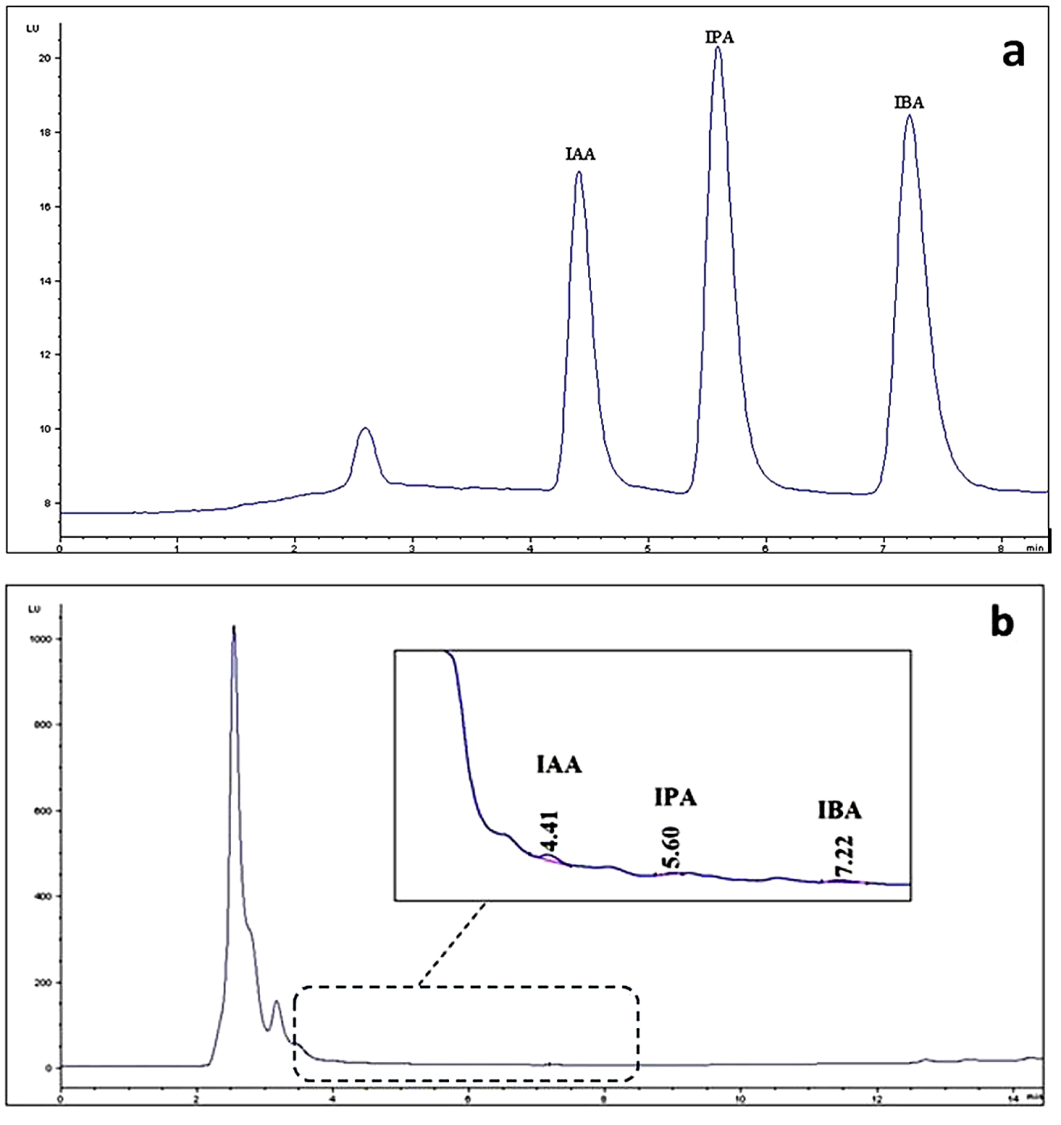
HPLC chromatogram of auxin standards and cyanobacterial materials; **a** HPLC chromatogram of auxin standards, **b** HPLC chromatogram of *Nostoc spongiaeforme* var. *tenue* ISB65 extract

Figure 3 displays the HPLC chromatograms of the intended auxin standards and a cyanobacterial sample. Furthermore, the concentrations calculated for the auxins in the cyanobacterial biomass are presented in Table 1. As summarized, IAA, IPA, and IBA are available in the *N. spongiaeforme* var. *tenue* ISB65 extracts with the predominance of IAA (35.3 ng.g^− 1^DW) followed by IPA (24.7 ng.g^− 1^DW) and IBA (4.2 ng.g^− 1^DW).

Table 2 represents the total N level (35.0 mg.L^− 1^), NH_4_^+^ (5.0 mg.L^− 1^), and NO_3_^−^ (9.4 mg.L^− 1^) in the cyanobacterial biomass, as well as the measurement methods. The amount of chemical content demonstrates the ability of cyanobacterial elicitors to increase critical mineral elements such as PO_4_^3−^, NO_3_^−^, NH_4_^+^, and total N in the culture media.

## Discussion

It is well known that the existence of soil microorganisms in the rhizosphere and their activity play a positive role in plants’ growth and productivity [38], and some strains of microorganisms, such as cyanobacteria, positively affect the vegetative and reproductive growth of medicinal plants in pot culture conditions [26, 32, 39, 40]. On the other hand, techniques such as in vitro culture of plants, as well as the application of biotic and abiotic elicitors, provide valuable methods for the improvement of secondary metabolite production in medicinal plants [13, 15, 22]. Given the scarcity of research on CL as a bioelicitor in medicinal plant tissue culture [41], this study examines the effects of CL on peppermint tissue culture.

The outcome of this study indicates that CL can significantly improve some growth indices in the treated plants under tissue culture conditions (Table 3). The results are congruent with the impact of cyanobacteria on the growth of potted plants, in which the ameliorating effect of cyanobacteria is attributed to N, phytohormones, and essential microelements providing features of these microorganisms [29, 31, 39, 42]. As seen in the analysis of cyanobacteria (Tables 1 and 2), similar to pot culture, the improvement of plant growth in vitro conditions and the increase in plant biomass can be attributed to the addition of hormones, i.e., IAA, IBA, and IPA, and non-hormonal plant growth-promoting substances, e.g., NO_2_^−^, NO_3_^−^, NH_4_^+^, and elemental cations such as K^+^, by cyanobacteria (Tables 1 and 2). Aside from the nitrogen-fixing ability of heterocystous *N. spongiaeforme* var. *tenue* ISB65, which results in growth enhancement of *Nostoc*-treated plants [32], the presence of cations such as K^+^ and Ca^2+^, as regulators of metabolic pathways and molecular activities of cells, in CL can result in the promotion of plant growth [26, 39]. Furthermore, phytohormones such as auxins, which are known for the induction and improvement of plant rooting and growth [43], and present in CL, are considered one of the main factors affecting the growth of treated plants.

In the present study, measurement of the plants’ photosynthetic pigments revealed an insignificant increase in chlorophyll and carotenoids in plants inoculated with CL compared to the control group (Table 4). Pigments are a principal index for the assessment of photosynthetic quality and quantity in various environmental conditions [44]. Some investigations have indicated that the addition of growth-promoting substances can increase the level of photosynthetic pigments [44, 45]. However, our investigation suggests that the presence of organic carbon in CL can alter the plant’s metabolism and prevent the enhancement of photosynthetic pigments [46].

The results of the current study showed that CL can also upregulate some metabolites of plants; *Nostoc*-treated plants (MS + CL culture medium) revealed a 2.3-fold increase in RA production (Table 5). Various factors, such as environmental factors, including photoperiod, moisture, temperature, pests, and disease, as well as the chemical content of culture media, could significantly alter the content of secondary metabolites in plants [47, 48]. The mentioned factors can directly or indirectly impact metabolic pathways and secondary metabolite production. Some of these factors are more effective in RA accumulation under cell and tissue culture conditions and are considered the key factors contributing to the final RA content in the tissue culture of medicinal plants. The most important factors affecting RA biosynthesis in medicinal plants are sucrose concentration [49], plant growth regulators such as auxins, cytokinin, and abscisic acid [21, 49], and the N source of the medium such as NO_3_^-^ and NH_4_^+^ concentrations [50]. 

Therefore, RA production in aromatic and medicinal plants can be optimized by upgrading cell and tissue culture methods, such as the addition of some precursors to culture media [51]. It seems that stimulation of some metabolite pathways can be the main factor in triggering the biosynthesis of RA and phenolic compounds [52]. Some microorganisms, as bioelicitors, can also be inoculated into plants for in vitro cultivation to stimulate the production of RA and phenolic compounds [53]. In this way, the enhancement of RA in transformed roots inoculated with microorganisms is attributed to signaling molecules, phytohormones, and stimulation of the biosynthetic pathways of key enzymes and their induced genes [54]. Moreover, an enhancement of total phenolic content in the inoculated roots can be linked to the Krebs cycle and plastid metabolism since the processes augment the amount of the precursors of phenolic compounds (e.g., fatty acids and carotenoids) and amino acids (e.g., tyrosine) [55]. Some researchers have introduced phytohormones such as cytokinin, abscisic acid, IBA, and 2,4-D as effective factors in stimulating the biosynthesis of RA and phenolic compounds under cell and tissue culture conditions in some plant species [21, 56, 57].

In this study, high concentrations of phytohormones such as IAA and IPA were observed in cyanobacterial biomass (Table 1). IBA was another phytohormone found in the CL (Table 1). Since there are many cyanobacteria with the ability to biosynthesize phytohormones, including abscisic acid, gibberellins, cytokinins, and auxins [30, 46], they have the potential to use purposefully to enhance some metabolites of plants, especially under in vitro conditions. 

In addition, an increase in the N sources of the culture medium by CL can be considered one of the main factors enhancing plant growth and the production of some plant metabolites. According to Ilieva and Pavlov, 1.2-fold improvement in NO_3_^−^ ion concentration in the cell culture of *Lavandula vera* results in an increase in RA concentration [50]. Rezasoltani et al. reported that the total nitrogen level of the extracts of the heterocystous cyanobacterium *Anabaena vaginicola* composes 10.63% of the cell dry weight [46].

The protective reactions of plants against several elicitors can stimulate the phenylpropanoid metabolic pathway, leading to the production of RA and free phenolic compounds [22]. The results of the current study reflected the ability of CL to induce growth and biomass production and to enhance RA content in peppermint. This increase can be attributed to the higher N source, auxin, and other mineral content of CL.

## Conclusions

In conclusion, the use of cyanobacterial bioelicitor under tissue culture conditions can increase plant growth, as well as the content of valuable phenolic compounds such as RA in *M. piperita.* Additionally, an enhancement in plant leaf number and biomass under tissue culture conditions is considered important, especially due to the economic value of this aromatic plant, as well as its application in food, cosmetic, and pharmaceutical products. Based on these results, the presence of phytohormones and the N content of CL can be regarded as the most important factors in altering metabolic pathways and improving RA production. Due to the economic implications of *M. piperita*, the use of a cyanobacterial bioelicitor is recommended under tissue culture conditions as a method for increasing RA production and possibly other valuable secondary metabolites.

## Data Availability

All data generated or analysed during this study are included in this published article.

## Abbreviations

DW: Dry weight
no.: Number(s)
RA: Rosmarinic acid
CL: Cyanobacterial lysate
MS: Murashige and Skoog
N: Nitrogen
NO_2_^−^: Nitrite
NO_3_^−^: Nitrate
NH_4_^+^: Ammonium
SO_4_^2−^: Sulfate
PO_4_^3−^: Phosphate
CO_3_^2−^: Carbonate
Mg^2+^: Magnesium ion
Ca^2+^: Calcium ion
Na^+^: Sodium ion
K^+^: Potassium ion
IAA: Indole 3-acetic acid
IBA: Indole 3-butyric acid
IPA: Indole 3-propionic acid
